# Venous thoracic outlet syndrome in the presence of cervical rib: report of two cases

**DOI:** 10.1590/1677-5449.202501112

**Published:** 2026-10-09

**Authors:** Juliana Borges Oliveira Cano, Talita Caroline de Oliveira Valentino, Alexandre Maierá Anacleto, Augustus Cézar Polimeno, Márcia Maria Morales

**Affiliations:** 1 Faculdade Ceres – FACERES, São José do Rio Preto, SP, Brasil.; 2 Hospital do Câncer de Barretos, Barretos, SP, Brasil.; 3 Universidade de São Paulo – USP, São Paulo, SP, Brasil.; 4 Universidade de Fortaleza – UNIFOR, Fortaleza, CE, Brasil.; 5 Universidade de São Paulo – USP, Ribeirão Preto, SP, Brasil.

**Keywords:** thoracic outlet syndrome, cervical rib, upper limb deep vein thrombosis, síndrome do desfiladeiro torácico, costela cervical, trombose venosa profunda do membro superior

## Abstract

Thoracic outlet syndrome is a rare condition caused by the compression of neurovascular structures in the region between the neck and chest. The venous form accounts for 2% to 3% of cases, with deep vein thrombosis most often caused by compression of the subclavian-axillary vein segment between the first rib and the clavicle. The occurrence of a cervical rib as a cause of venous deep vein thrombosis is even rarer. In this report, we present the diagnosis, treatment, and outcome of 2 cases of venous deep vein thrombosis resulting from 2 different mechanisms. The approach was based on a review of the literature on venous thoracic outlet syndrome and cervical rib.

## Resumo

A síndrome do desfiladeiro torácico (SDT) é uma condição rara causada pela compressão das estruturas neurovasculares na região entre o pescoço e o tórax. A forma venosa é responsável por 2 a 3% dos casos, sendo a trombose venosa profunda (TVP) mais comumente causada pela compressão do segmento da veia subclávia-axilar entre a primeira costela e a clavícula. A ocorrência de uma costela cervical como causa de TVP venosa é ainda mais rara. Neste relato, apresentamos o diagnóstico, o tratamento e o desfecho de dois casos de TVP resultantes de mecanismos diferentes. A abordagem foi baseada em uma revisão da literatura existente sobre a SDT venosa e a costela cervical.

## INTRODUCTION

Thoracic outlet syndrome (TOS) is a rare clinical abnormality caused by compression of the brachial plexus, subclavian artery, and subclavian vein in the thoracic outlet,^1^ a space bounded by the clavicle, first rib, costoclavicular ligament, and posterior scalene.^2,3^ It involves the scalene triangle, the costoclavicular space, and the pectoralis minor space.^4,5^

Neurological compression is the most common type, accounting for more than 95% of cases.^6^ In most cases, deep vein thrombosis (DVT) is caused by compression between the first rib and the clavicle.^1,2^ Risk factors include biotypes and individual variations, such as genetics, age, and sex.^4,7^

In addition, effort-induced thrombosis, also known as Paget–Schroetter syndrome,^8^ is the most relevant triggering factor for upper extremity DVT in this context. It typically occurs in young and otherwise healthy individuals following repetitive or strenuous upper limb activity, leading to endothelial injury and thrombosis in the setting of pre-existing anatomic compression.

Rarely, cervical ribs, anomalies of the clavicle and first rib act on the mechanism of compression and venous injury. Among these anomalies, cervical rib is the most common, occurring bilaterally in approximately 50% of cases. Its prevalence in the general population ranges from 0.27% to 0.74%, and it is 2 to 3 times more frequent in women. The presence of bone abnormalities as a causative factor in TOS, which is more evident in the neurogenic and arterial forms, is rarely associated with venous TOS.^9^

This study describes the cases of 2 patients who presented with venous thrombosis of the left axillary-subclavian transition in the presence of a cervical rib. Their clinical characteristics, imaging findings, management and outcomes are described below.

The research ethics committee of the involved institution approved this study (decision 7.469.761), and the patients provided written consent for the publication of this case report.

## CASE 1

A previously healthy 29-year-old female medical student developed subclavian vein thrombosis, which was treated with 6 months of anticoagulation therapy. No underlying anatomical cause was identified at that time, delaying definitive management.

For the next 4 years she remained symptomatic, with mild edema and discomfort of the left upper limb during routine activities, consistent with McCleery syndrome,^10^ which prompted referral to vascular surgery.

On examination, there was mild collateral circulation in the anterior chest wall, slight swelling, and discomfort with left arm hyperabduction.

Diagnostic tests included: 1) X-ray of the cervical spine, which revealed bilateral cervical ribs (Figure 1); 2) computed tomography of the costal arches with 3-dimensional reconstruction, which further corroborated bilateral cervical ribs (Figure 2); 3) arterial and venous Doppler ultrasound of the subclavian and axillary vessels at rest and with hyperabduction maneuvers, which showed subclavian artery with turbulent flow and a significant increase in velocity at its intersection with the clavicle during limb abduction at 90º and an absence of distal flow at 120º, in addition to a recanalized subclavian vein, with a thin fibrous traverse, distal segment of the subclavian vein and a patent axillary vein; 4) angiotomography of the subclavian vessels at rest and in hyperabduction maneuvers, during and after intravenous injection of the contrast medium, which showed the cervical rib bilaterally in the plane of the middle scalene muscle and posteriorly to the subclavian artery and brachial plexus, with the cervical rib causing venous compression during hyperabduction maneuvers, being outside the costoclavicular space; the posterior scalene muscle was slightly prominent, protruding anteriorly in relation to the subclavian artery and postero-inferiorly in relation to the subclavian vein (Figure 3). Laboratory tests for hereditary and acquired thrombophilias were normal.

**Figure 1:**
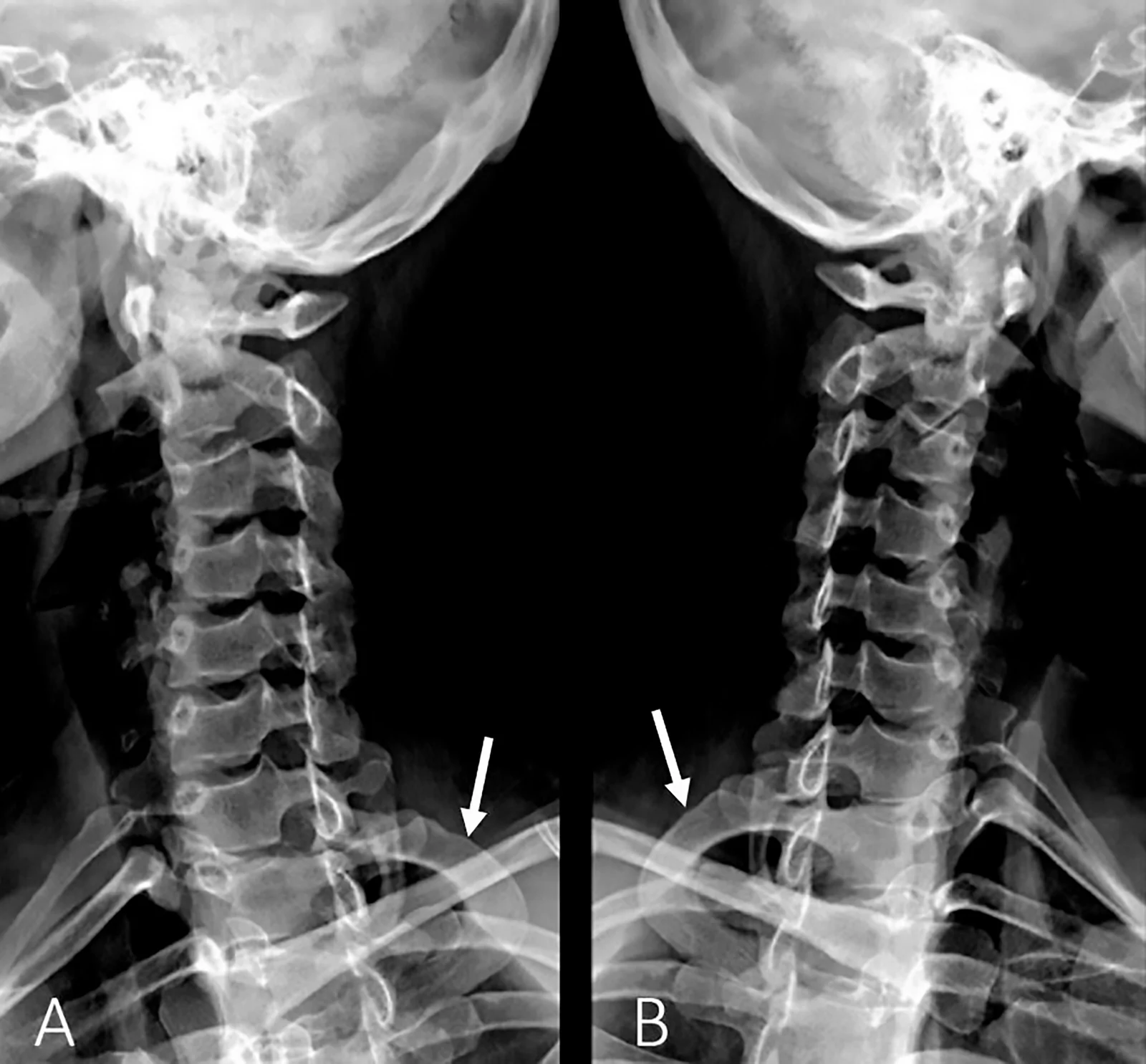
X-ray of the cervical spine: (A) right anterior oblique view; (B) left anterior oblique view, showing rectification of the physiological cervical curvature, bilateral presence of a complete cervical rib (arrows), and preserved intervertebral spaces.

**Figure 2:**
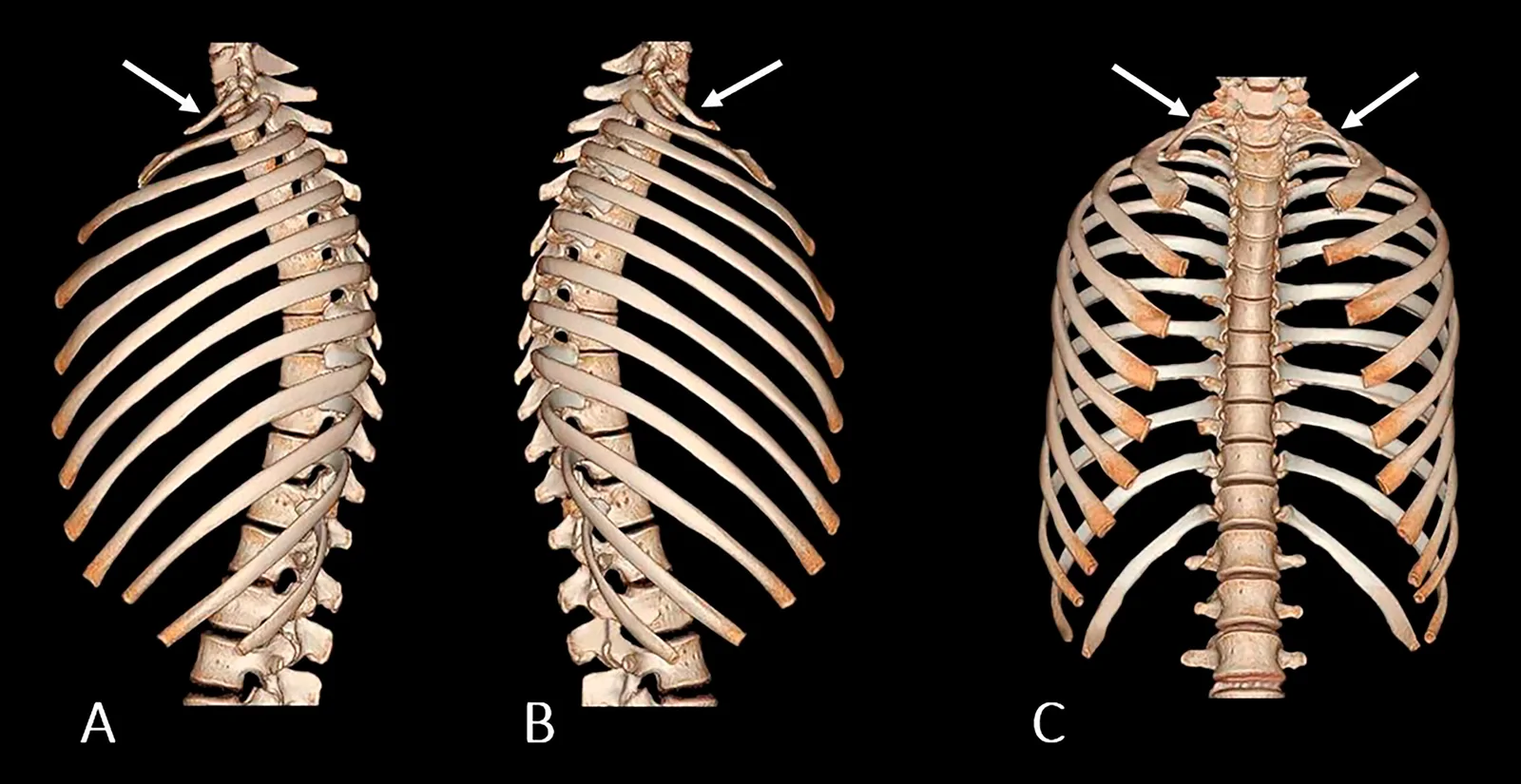
Computed tomography scan of the rib arches with 3-dimensional reconstruction: (A) left lateral view; (B) right lateral view; (C) anteroposterior view with complete bilateral cervical ribs (arrows) and preserved sternoclavicular joint spaces and surfaces.

**Figure 3:**
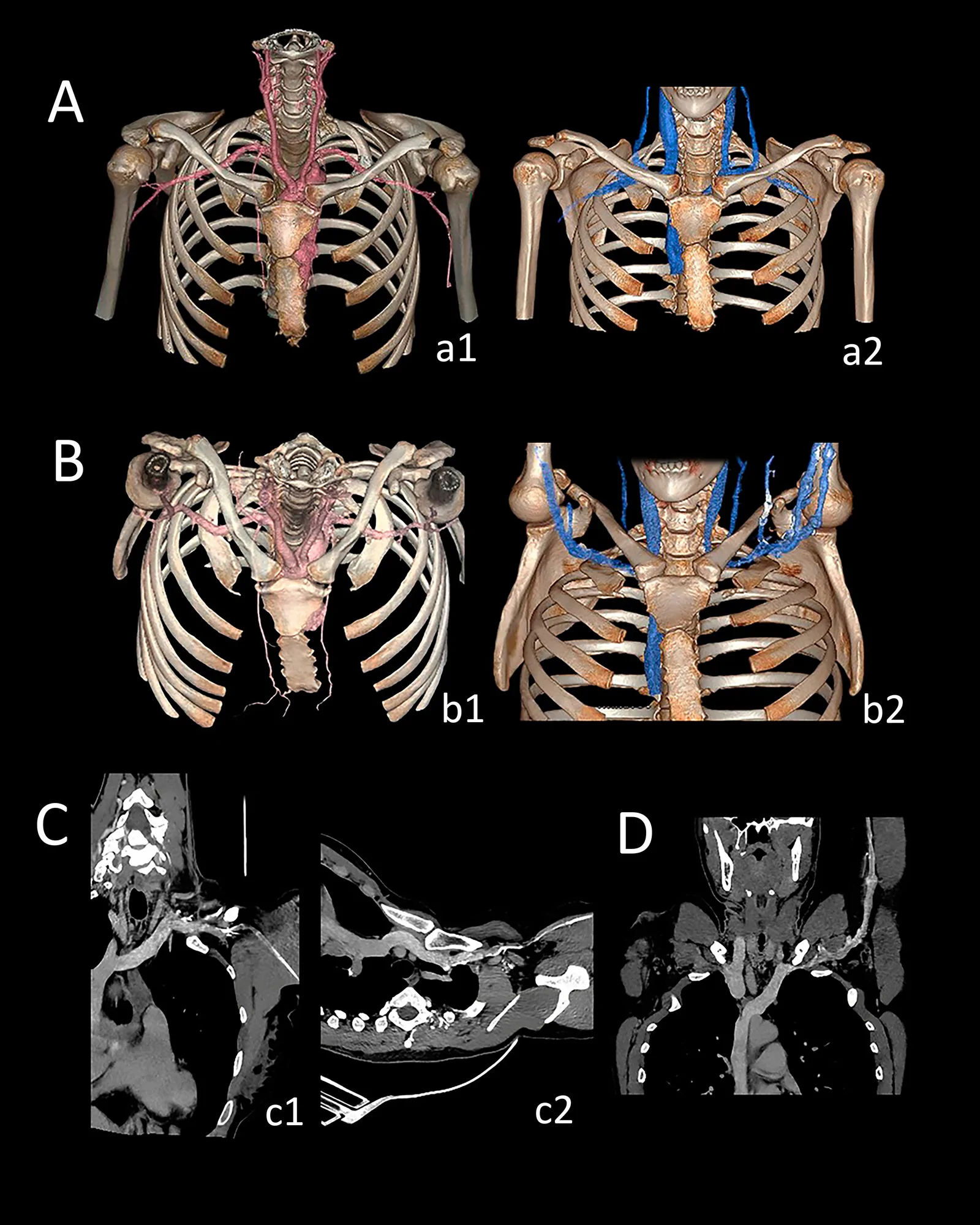
Angiotomography of subclavian vessels with recording of venous and arterial phases at rest and with hyperabduction maneuvers: (A) image at rest with reconstruction of the subclavian-axillary transition in the arterial (a1) and venous (a2) phases; (B) image with hyperabduction maneuver with reconstruction of the subclavian-axillary transition in the arterial (b1) and venous (b2) phases; (C) axial section (c1) and coronal section (c2) of the left subclavian vein at rest; (D) coronal section of the left subclavian vein under stress.

The proposed treatment was cervical rib resection via supraclavicular access, with intraoperative reassessment of the costoclavicular space, as imaging suggested venous compression by the cervical rib rather than the costoclavicular space, both at rest and during hyperabduction.

The patient was positioned supine with the back elevated 30°, using an interscapular cushion for cervical extension and right rotation. The left upper limb was prepared in the operative field. A supraclavicular transverse incision was made, followed by subplatysmal dissection. The omohyoid was sectioned, exposing the anterior scalene muscle. The phrenic and long thoracic nerves were identified and preserved. An anterior scalenectomy was performed by dissecting its insertion at the first rib, protecting the phrenic nerve, subclavian artery, and brachial plexus roots (C5–T1). The plexus was mobilized medially, exposing and resecting the middle scalene insertion. The cervical rib, located posterior to the plexus and subclavian artery, was identified, sectioned, and disarticulated from the first rib.

After resection, the costoclavicular space was tested with the Falconer-Weddell maneuver, confirming no compression. Hemostasis was secured, the wound drained, and closure completed (Figure 4). No motor deficits, pneumothorax, or diaphragmatic elevation occurred. The patient was discharged on postoperative day 2, having received guidance about supervised rehabilitation.

**Figure 4:**
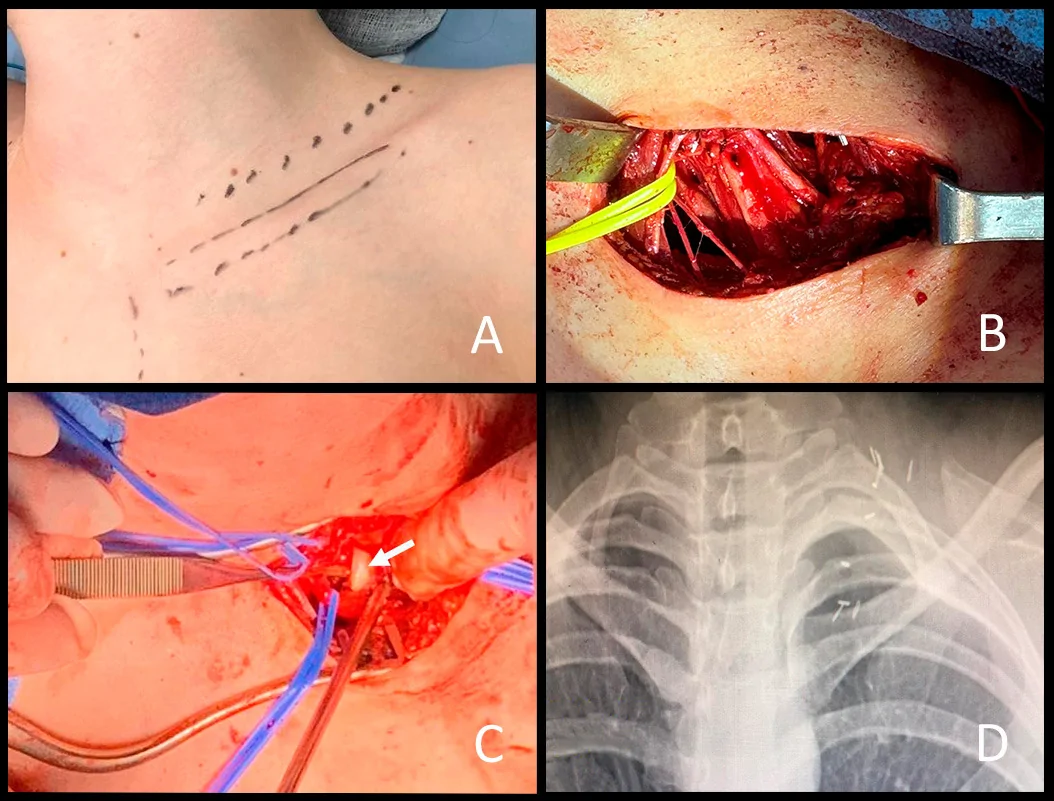
Intraoperative resection of the cervical rib by supraclavicular access: (A) dermal marking for supraclavicular access; (B) roots of the brachial plexus identified and preserved; (C) left cervical rib; (D) control x-ray after resection of the left cervical rib.

Short-term oral anticoagulation with rivaroxaban 20 mg daily was prescribed for 30 days. After 34 months, she remains symptomatically improved without recurrent thrombosis.

## CASE 2

A 31-year-old woman presented with acute pain, edema, and cyanosis the left upper limb. Doppler ultrasound confirmed left subclavian vein occlusion (mid-distal). Hyperabduction maneuvers showed reduced right subclavian venous phasicity, decreased right subclavian arterial flow, and interrupted left arterial flow. Phlebography (Figure 5) confirmed the diagnosis, which was followed by catheter-directed thrombolysis with alteplase, heparinization, and oral anticoagulation.

**Figure 5:**
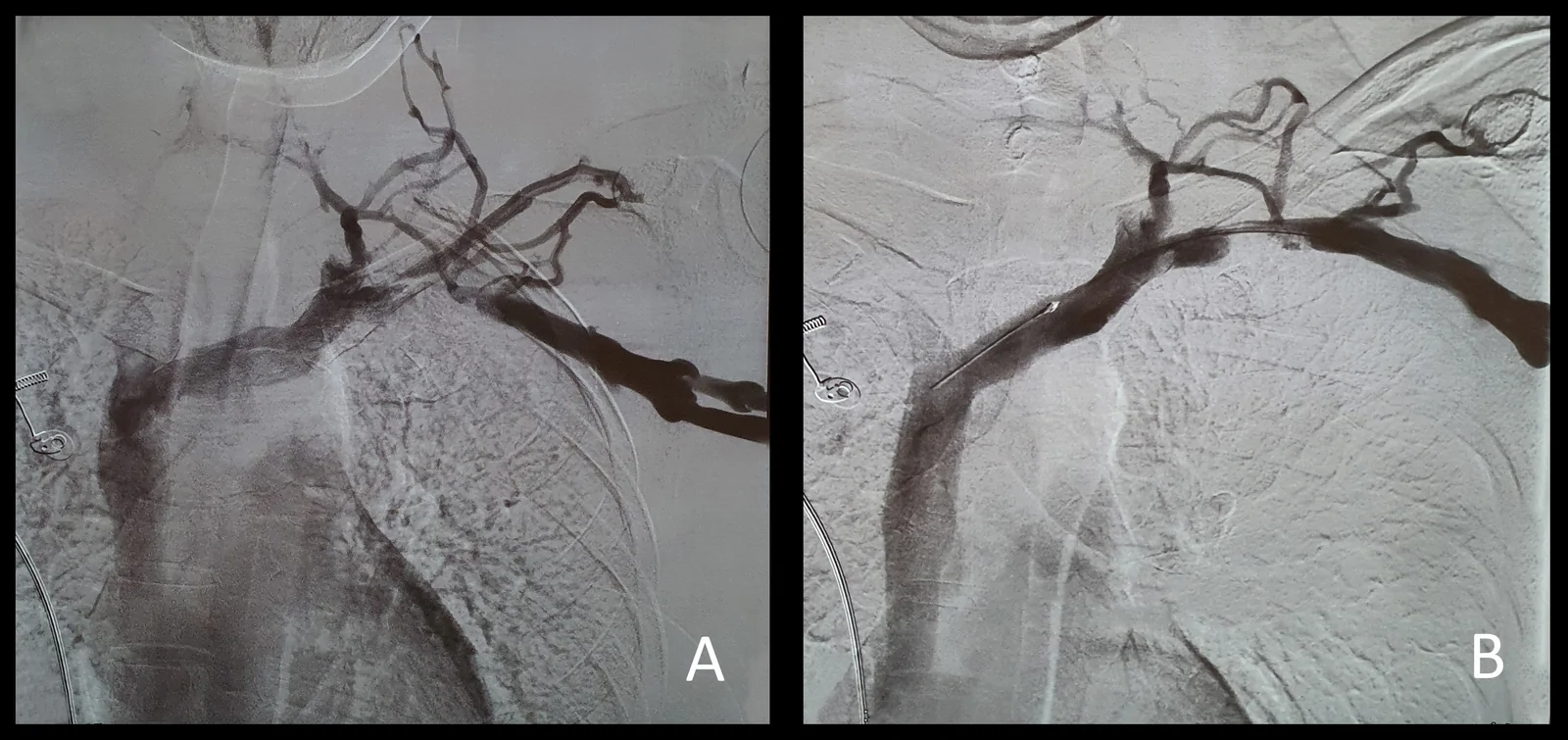
Upper limb phlebography confirming acute thrombosis of the left subclavian vein: (A) catheter thrombolysis using alteplase (B).

The etiological investigation included: 1) X-ray of the cervical spine, which revealed rudimentary cervical ribs (Figure 6); 2) chest tomography with 3-dimensional reconstruction of the rib arches, which showed rudimentary cervical ribs; 3) angiotomography of the subclavian vessels with arterial and venous phases, at rest and during hyperabduction maneuvers of the upper limbs (Figure 7); 4) normal laboratory tests for hereditary and acquired thrombophilias in the acute thrombosis phase. Three weeks after the catheter-guided thrombolysis, surgical resection of the cervical rib and the first rib by paraclavicular access was scheduled.

**Figure 6:**
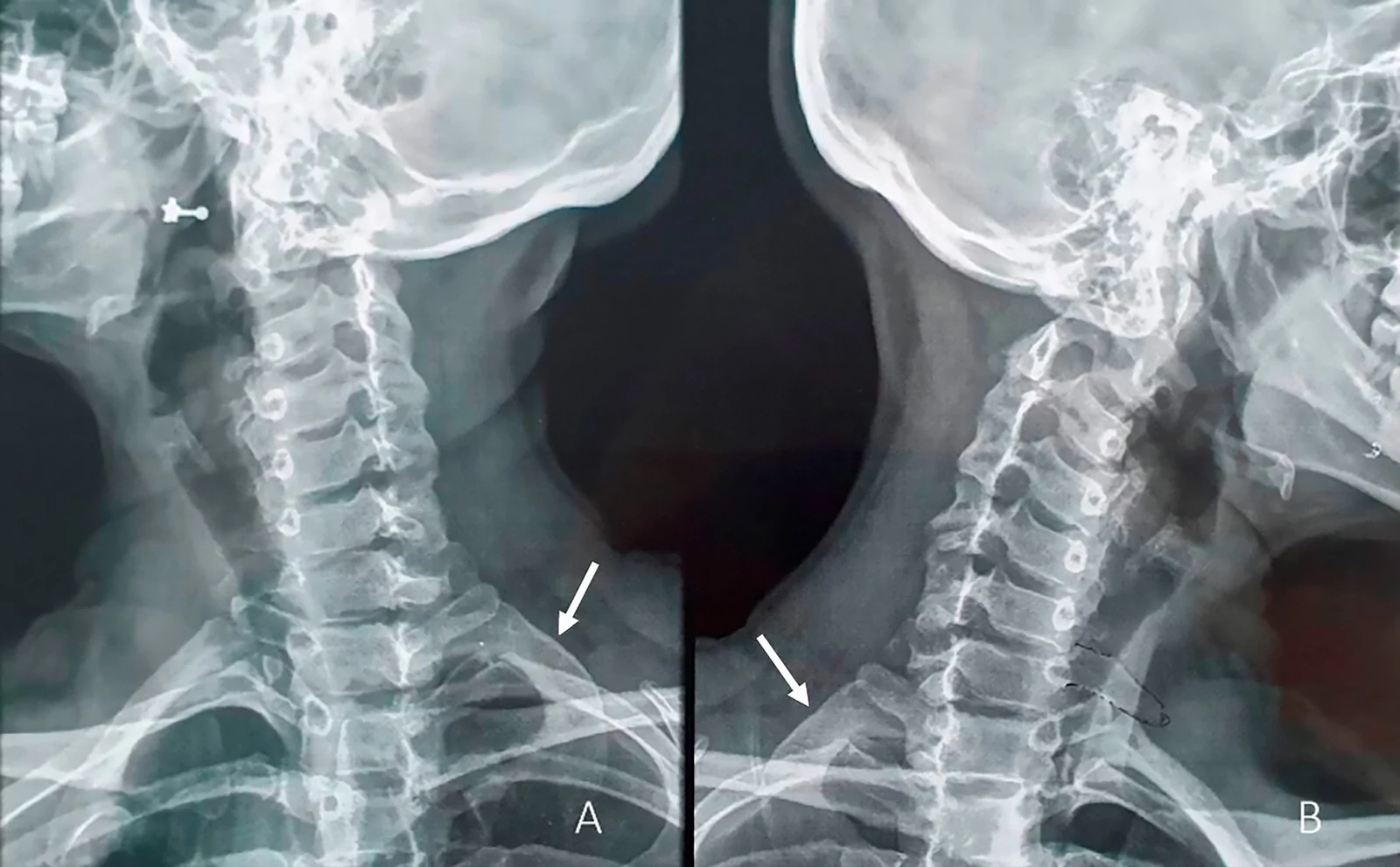
X-ray of the cervical spine: (A) right anterior oblique view; (B) left anterior oblique view showing preserved cervical axis, cervical ribs (arrows), and preserved intervertebral spaces.

**Figure 7:**
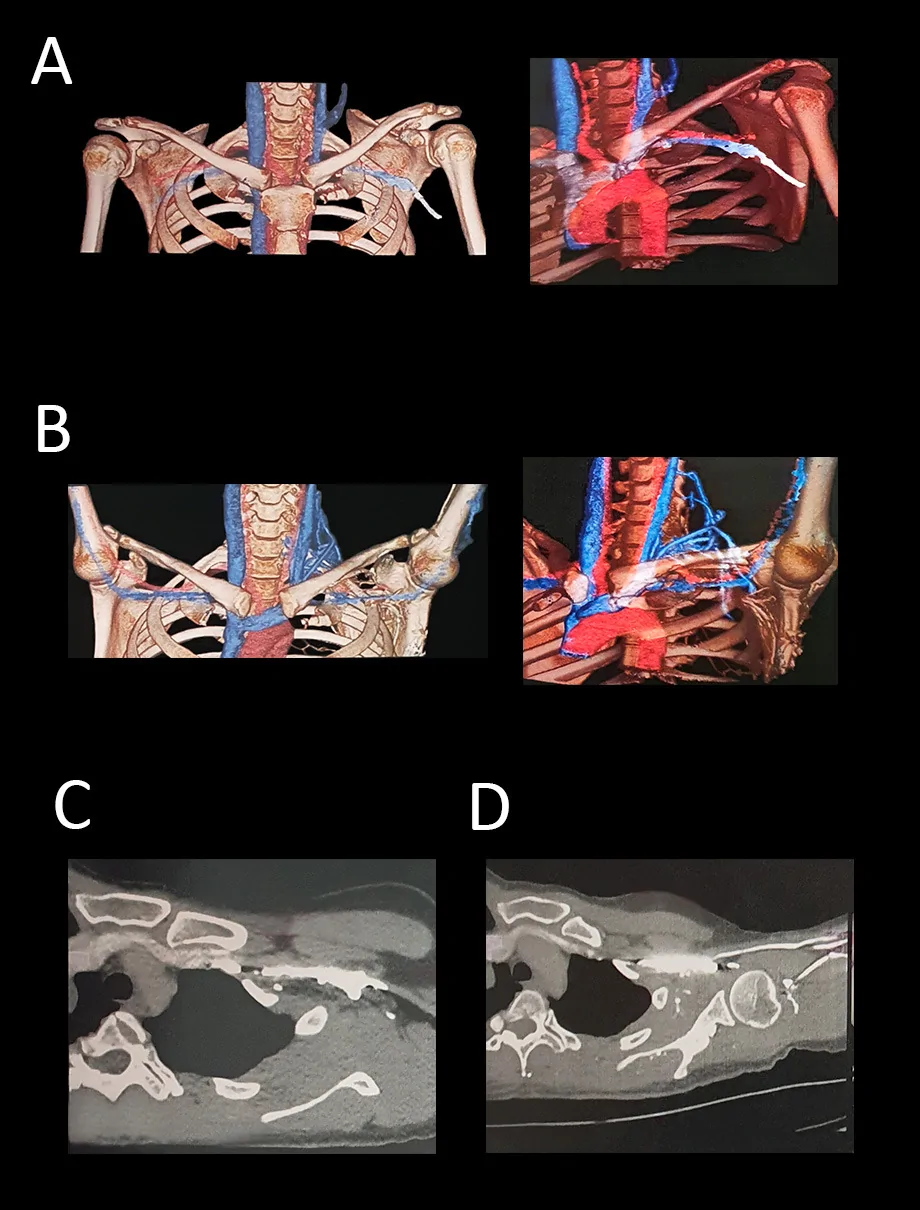
Angiotomography of subclavian vessels with the recording of venous phase at rest and during hyperabduction maneuvers: (A) resting image of bilateral complete cervical rib, distal ends with posterior projection in relation to the neurovascular bundle; (B) stress image of the bilateral complete cervical rib, distal ends with posterior projection in relation to the neurovascular bundle; (C) section of the left subclavian vein at rest; (D) section of left subclavian vein under stress.

The patient was positioned as in Case 1. Paraclavicular access was chosen for resection of the rudimentary cervical and first ribs due to possible venous reconstruction. A supraclavicular incision with dissection, as in Case 1, was combined with an infraclavicular incision extending from the manubrium to the deltopectoral groove. The platysma and pectoral fascia were opened, and the pectoral muscle was separated to expose the first rib. The anterior and middle scalene muscles were detached, the intercostal muscles were sectioned, the subclavius muscle was resected, and the pleura was dissected. The first rib was also resected. The subclavian-axillary venous transition was inspected, requiring only external venolysis without reconstruction. Hemostasis was secured, pleural integrity checked, and the wound was drained and closed (Figure 8). There were no postoperative motor deficits or pneumothorax. The patient was discharged on day 2, having received guidance about supervised rehabilitation. After 24 months, the symptoms improved, with no recurrence of thrombosis. Short-term oral anticoagulation with rivaroxaban 20 mg daily was prescribed for 90 days.

**Figure 8:**
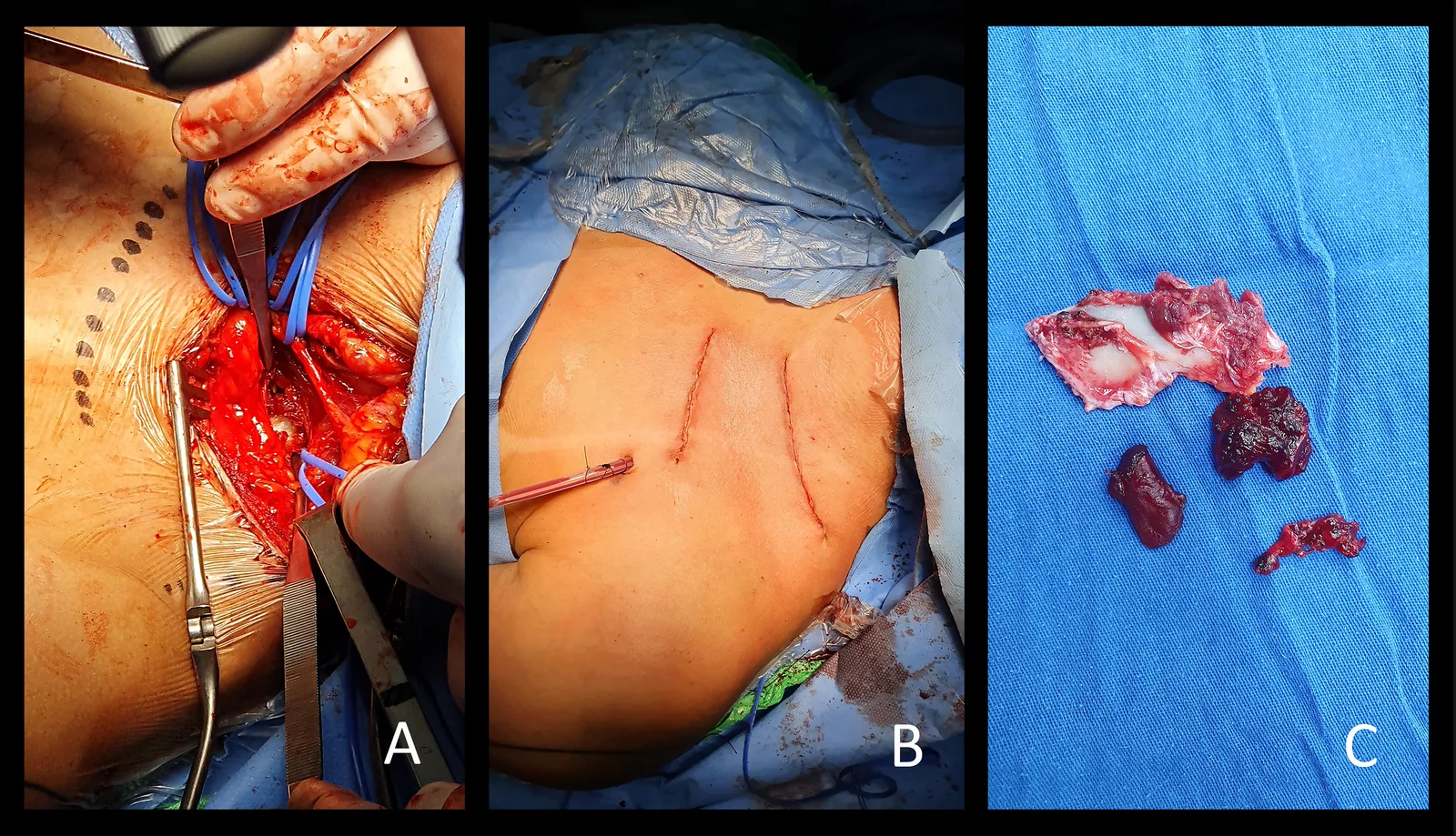
Intraoperative resection of the rudimentary cervical rib and first rib by paraclavicular access: (A) paraclavicular incision with access to the rudimentary cervical rib and first rib; (B) final aspect after resection of the rudimentary cervical rib and first rib; (C) result of resection of the left first rib and subclavius muscle.

## DISCUSSION

This report describes 2 clinical cases of DVT due to compression of the vein at the left subclavian-axillary transition: the first due to the presence of a cervical rib and the second due to compression in the costoclavicular space.

Ghefter et al.^11^ define the cervical rib as an anomalous rib arising in the transverse process of C7 or as a first rib arising in the transverse process of T1, reporting their incidence as approximately 0.76 and 0.74%, respectively. Cervical ribs are incomplete in 70% of cases, but almost always involve fibrous fusion with the first rib. Of this population, 10% have related symptoms. Table 1 below describes the 4 varieties of cervical rib.

**Table 1:** Description of anatomical aspects of the cervical rib.

**Type**	**Characteristics**
I	Complete, it articulates in the first rib or the sternal manubrium.
II	Incomplete, it has an expanded bulb-shaped end.
III	Incomplete, joined to the first rib by a fibrous band.
IV	Rudimentary, small bone portion slightly larger than the transverse process of C7.

Source: Ghefter et al.^11^

Neurological compression is the most common form of TOS (90%).^12^ Arterial complications, though rare, usually result from subclavian artery compression by a complete cervical rib, while venous complications stem from subclavian vein compression due to anatomical variations.^1^

Venous symptoms are characterized by edema and cyanosis of the affected upper limb.^11^ It is worth noting that the venous form accounts for only 2% to 3% of cases,^7^ and Weber and Criado^9^ point out that the incidence of subclavian-axillary venous thrombosis caused by the presence of a cervical rib is 2%, based on a retrospective analysis of 400 patients, 115 of whom had TOS due to bone anomalies and 80 of whom had a cervical rib. Masocatto et al.^1^ reported that the incidence of this syndrome varies from 3 to 80 cases per 1,000 people. While the neurogenic form accounts for around 90% of TOS cases, the venous form accounts for between 3% and 5% and the arterial form for < 1%. The incidence of the venous form is 1:100,000 people per year and is more common in men. The typical age of involvement for the vascular forms is between 20 and 30 years, while that of the neurogenic form is between 20 and 40 years, being more common in women. There is no sex prevalence for the arterial form.^1^

In suspected upper-limb DVT, imaging is essential.^13,14^ Venous Doppler ultrasound is the first choice for diagnosis and etiological investigation. Plain cervical and chest radiographs help detect bone anomalies. Although these anomalies are associated with arterial or neurogenic TOS, they can also be seen in patients with venous syndrome. In addition, magnetic resonance venography and computed tomographic venography are useful when symptoms are atypical or duplex results inconclusive.^15^

TOS management depends on pathology and presentation. In venous compression with a cervical rib, resection is the treatment of choice, and it is also important to assess the need for concomitant resection of the first rib.

The choice of a paraclavicular approach in Case 2 was based on the venous etiology and the site of compression. Unlike Case 1, the symptoms were related to costoclavicular compression involving the first rib rather than the rudimentary cervical rib, requiring first rib to be resected in addition to cervical rib.

In venous thoracic outlet syndrome, decompression is more effectively achieved through infraclavicular or combined approaches, as supraclavicular access alone provides limited exposure of the subclavian–axillary vein. Furthermore, given the history of effort thrombosis, direct venous access for possible reconstruction was anticipated, favoring a paraclavicular approach, although reconstruction was ultimately not required.

Anticoagulation alone is associated with suboptimal outcomes in venous thoracic outlet syndrome.^16^ Thrombolysis represents a significant advancement in acute cases, allowing rapid symptom relief, restoration of venous patency, and identification of the underlying cause of thrombosis. When followed by surgical decompression, it provides superior long-term results.^17^ Current evidence supports offering thrombolysis to patients presenting with acute DVT within the first 2 weeks.

The decision for surgical or non-surgical management in venous thoracic outlet syndrome should be individualized, considering the patient’s age, activity level, comorbidities, and functional status. Although surgical decompression carries risks, including pain, bleeding, and nerve injury, long-term outcomes are favorable.

A staged approach is commonly recommended: thrombolysis in acute cases (within 14 days), followed by anticoagulation and reassessment. Surgical decompression is indicated in patients with persistent symptoms, significant extrinsic venous compression, or recurrent thrombosis, with adjunctive endovascular or reconstructive procedures as needed (within 90 days).^18^ Debate persists regarding when to perform decompression surgery after thrombolysis in patients with primary upper extremity deep vein thrombosis. Further research comparing various intervention schedules is essential to determine the best clinical timeline.^19^

Conservative management carries the risk of DVT recurrence, while surgery is highly effective and has low morbidity.

## CONCLUSIONS

The individualized management of DVT caused by subclavian–axillary vein compression due to a cervical rib was a key differential in this report. The cases illustrate distinct anatomical mechanisms requiring tailored surgical approaches, underscoring the importance of a multidisciplinary, personalized strategy in treating this rare condition.

## Data Availability

Research data is available in the body of the article.
